# Complex Fatal Self-Harm Under Psilocybin Intoxication: Suicidal Manner of Death or Not?

**DOI:** 10.1177/19253621261468084

**Published:** 2026-08-21

**Authors:** Caleb Labonté, Alfredo E. Walker

**Affiliations:** 1 Department of Pathology and Laboratory Medicine, 6363 University of Ottawa, The Ottawa Hospital General Campus, Ottawa, Ontario, Canada; 2 Department of Pathology and Laboratory Medicine, 6363 University of Ottawa, The Ottawa Hospital General Campus, Ottawa, Ontario, Canada

**Keywords:** Complex suicide, Psilocybin, Asphyxia, Hanging

## Abstract

Psilocybin is a hallucinogen that causes significant changes in perception, mood, and cognition. Challenging effects include “bad trips” with the risk of physical harm, enduring psychological symptoms, and self-harming attempts. Previous reports of simple fatal self-harming with psilocybin ingestion have been described with methods that include self-inflicted wounds and self-initiated descent from a height. Complex fatal self-harming that employs more than one method is uncommon in forensic pathology and has not been previously associated with psilocybin use. A case of complex fatal self-harming following psilocybin ingestion in an individual with no history of suicidal ideation or attempts is reported. Findings at the scene and autopsy were consistent with self-inflicted incised wounds and plastic bag asphyxia-facilitated hanging in the context of use of magic mushrooms. The urine was positive for psilocin, the metabolite of psilocybin, and the quantification of psilocybin in the blood failed due to analytical issues. This case highlights the potential harm of psilocybin ingestion, the resultant complex pattern of fatal self-inflicted injury that can occur, and the challenge to certify suicide as the most appropriate manner of death when no pre-existent intent to fatally self-harm exists.

## Introduction

Psilocybin is a hallucinogen found in more than 200 species of basidiomycetes fungi, commonly known as “magic mushrooms,” which are distributed worldwide and subject to legal restrictions due to recreational usage.^1^ Although it exhibits low toxicity, it mediates significant changes in perception, mood and cognition through agonism with the 5-HT2A receptor.^2–4^ It has gained increased attention for its potential application in the treatment of psychiatric disorders including treatment-resistant depression, anxiety, substance use disorder, and obsessive-compulsive disorder.^5^ However, a systematic review revealed that the association between suicidality and psychedelic use in both clinical and recreational settings is mixed.^6^

Challenging effects associated with psilocybin use include “bad trips” that present with a risk of physical harm, enduring psychological symptoms and attempted suicide.^7^ Previous reports of suicide under the influence of psilocybin are rare and include self-inflicted wounds and falls from a height.^8–11^ Importantly, completed self-harming and self-harming attempts have been described in individuals without a prior psychiatric history.^9,12^

Suicides are classified as simple when only one method of self-harming is employed, or complex when more than one self-harming method is utilized. Complex suicides are uncommon occurrences in forensic pathology and are estimated to represent 1.5–5% of all completed suicides.^13^ To the best of our knowledge, there is no previously published case of complex fatal self-harming under the influence of psilocybin.

We report the first case of complex fatal self-harm in the setting of psilocybin ingestion to highlight the potential for a varied presentation of fatal self-inflicted injury under the influence of psilocybin compared to previous reports. The absence of a history of suicidality in the decedent underscores the risk of fatal self-harm associated with psilocybin use and the challenge to appropriately certify the manner of death when a demonstrated intent to fatally self-harm cannot be established.

## Case Report

**Figure 1.**
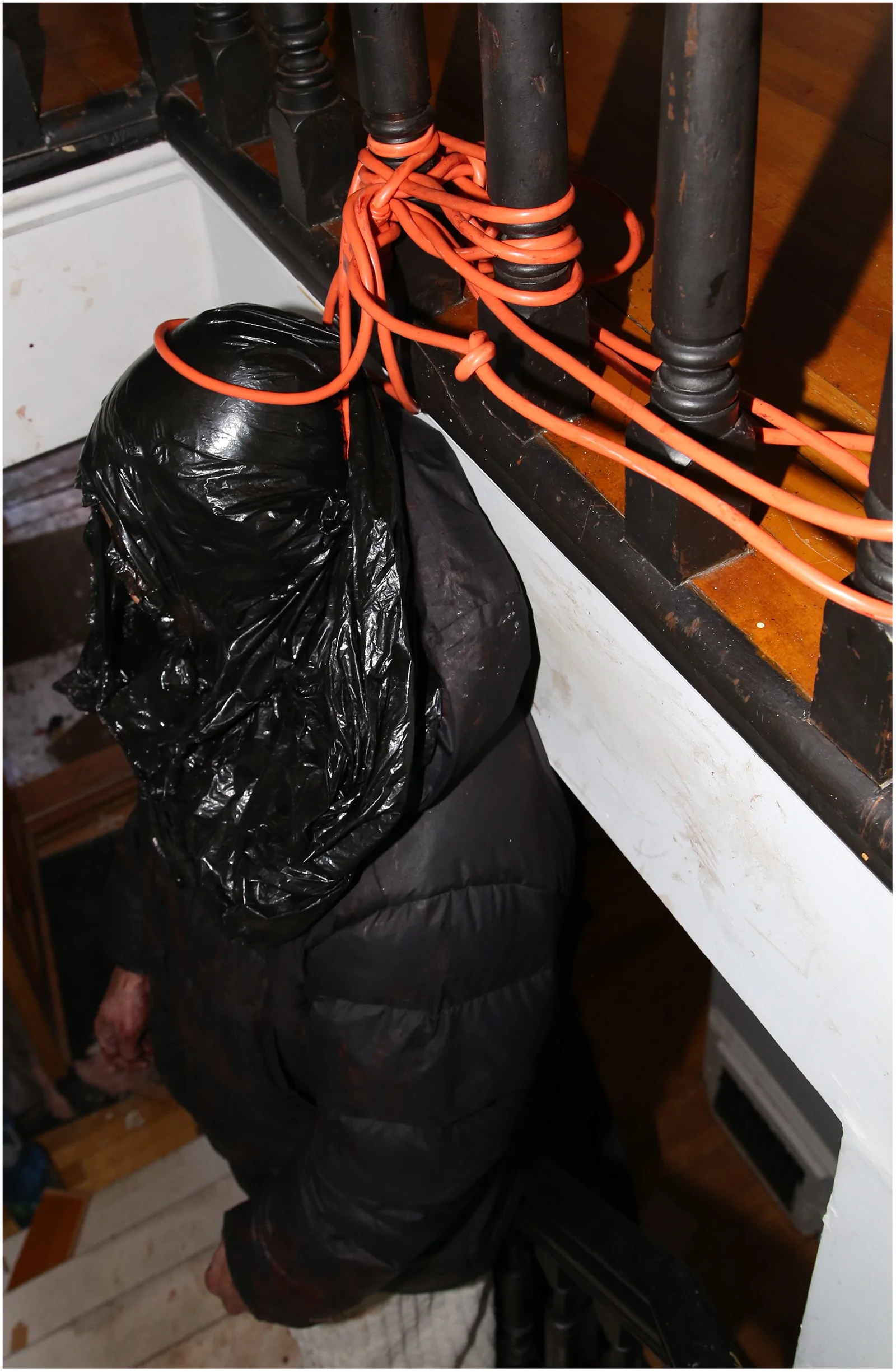
The body suspended from the rungs of the banister by an electrical extension cord, with the mouth of a plastic bag occluded by the electrical cord ligature around the decedent's neck.

**Figure 2.**
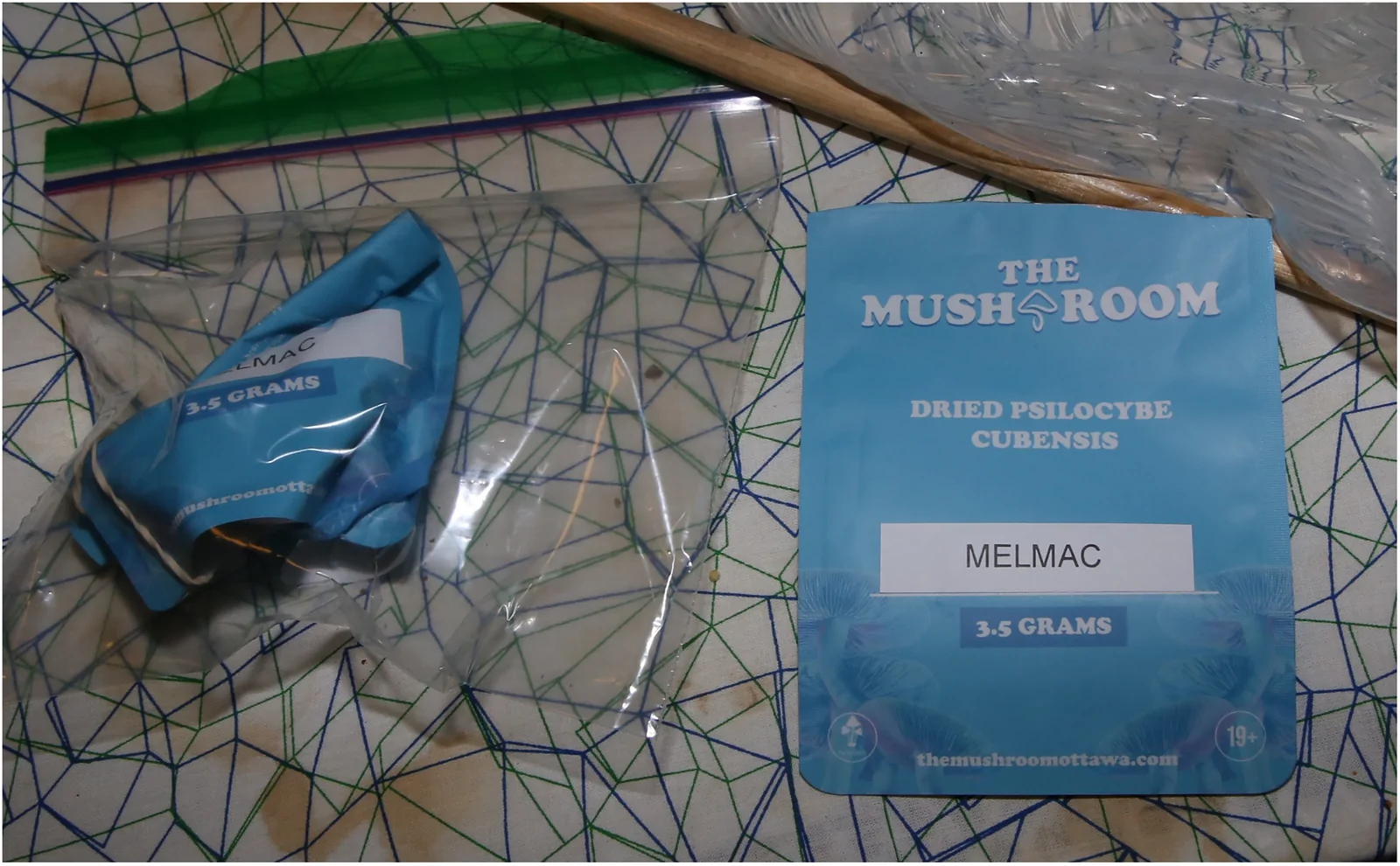
Magic mushroom on the scene.

**Figure 3.**
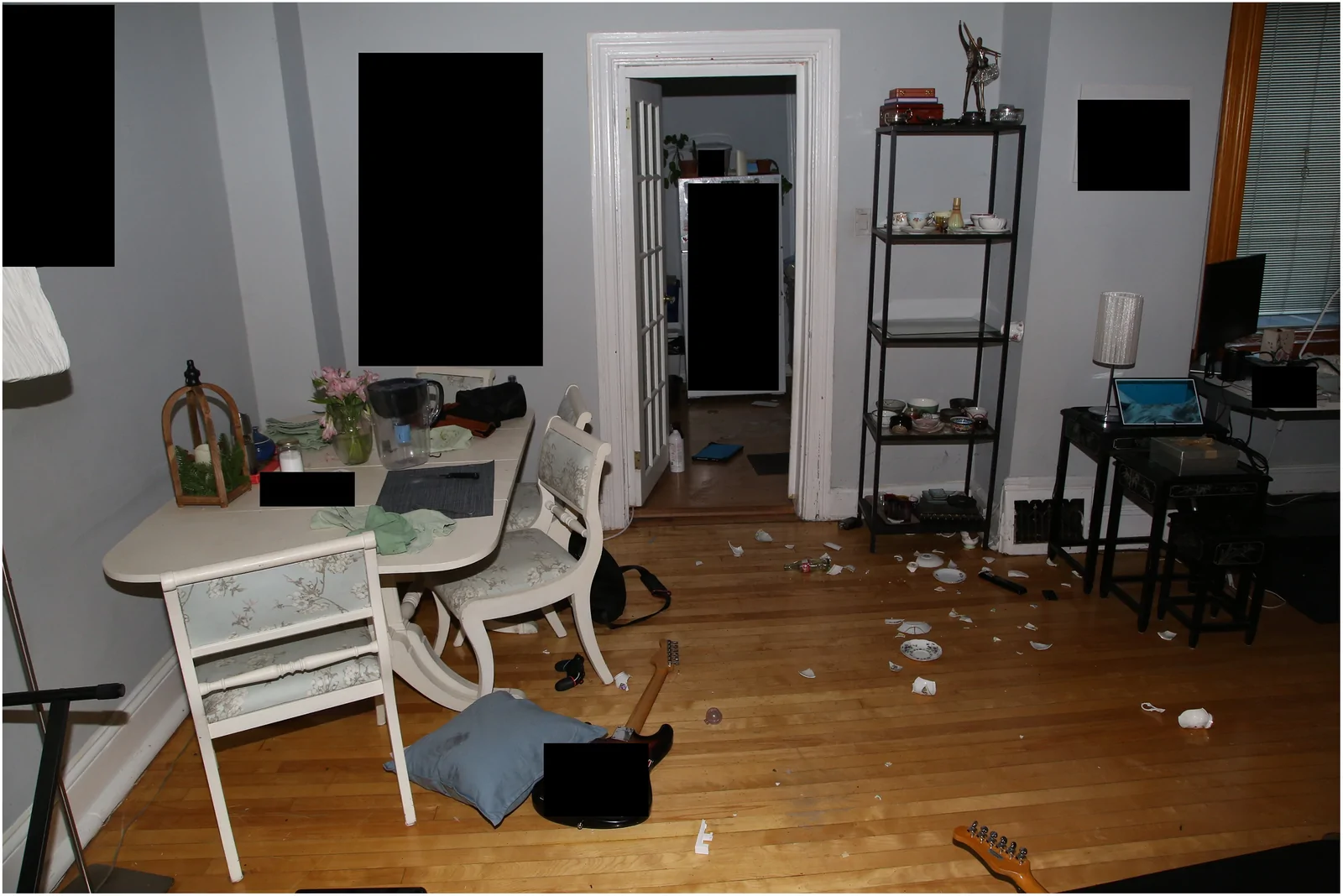
Disarray of the first floor with broken dishes.

The decedent was a 32-year-old man with a history of obsessive-compulsive disorder, trichotillomania, and misuse of cannabis and magic mushrooms, but no history of suicidal ideation or attempts. He had reportedly advised his girlfriend of his intent to use magic mushrooms alone while she was away on vacation. After missing a phone call, a friend was sent to confirm his well-being at the behest of his girlfriend. The friend observed the decedent hanging from the stairs in his secure apartment and called the police. Upon their arrival, the doors to the residence were locked. The first floor of the house was in disarray with overturned furniture and broken dishes on the floor. Magic mushroom packages were in the kitchen and marijuana was in the pantry. There was abundant blood staining in the upstairs bathroom with a blood-stained knife on the counter. The toilet was filled with an opaque liquid. An empty bottle of liqueur, a container of bleach, and a container of orange juice were on the bathroom floor. The dressed body was suspended over the stairs from the banister, a few centimeters above a step, by an electrical extension cord around the neck as a cervical ligature. A black plastic garbage bag was over the decedent's head, with the mouth of the bag occluded by the electrical cord ligature that was around the decedent's neck on the outside of the bag. The items of clothing worn consisted of a winter jacket and a bloodstained white towel around the hips. Multiple incised wounds of the anterior aspects of both forearms were evident. The electrical cord ligature was focally bloodstained (Figures 1 to 3).

**Figure 4.**
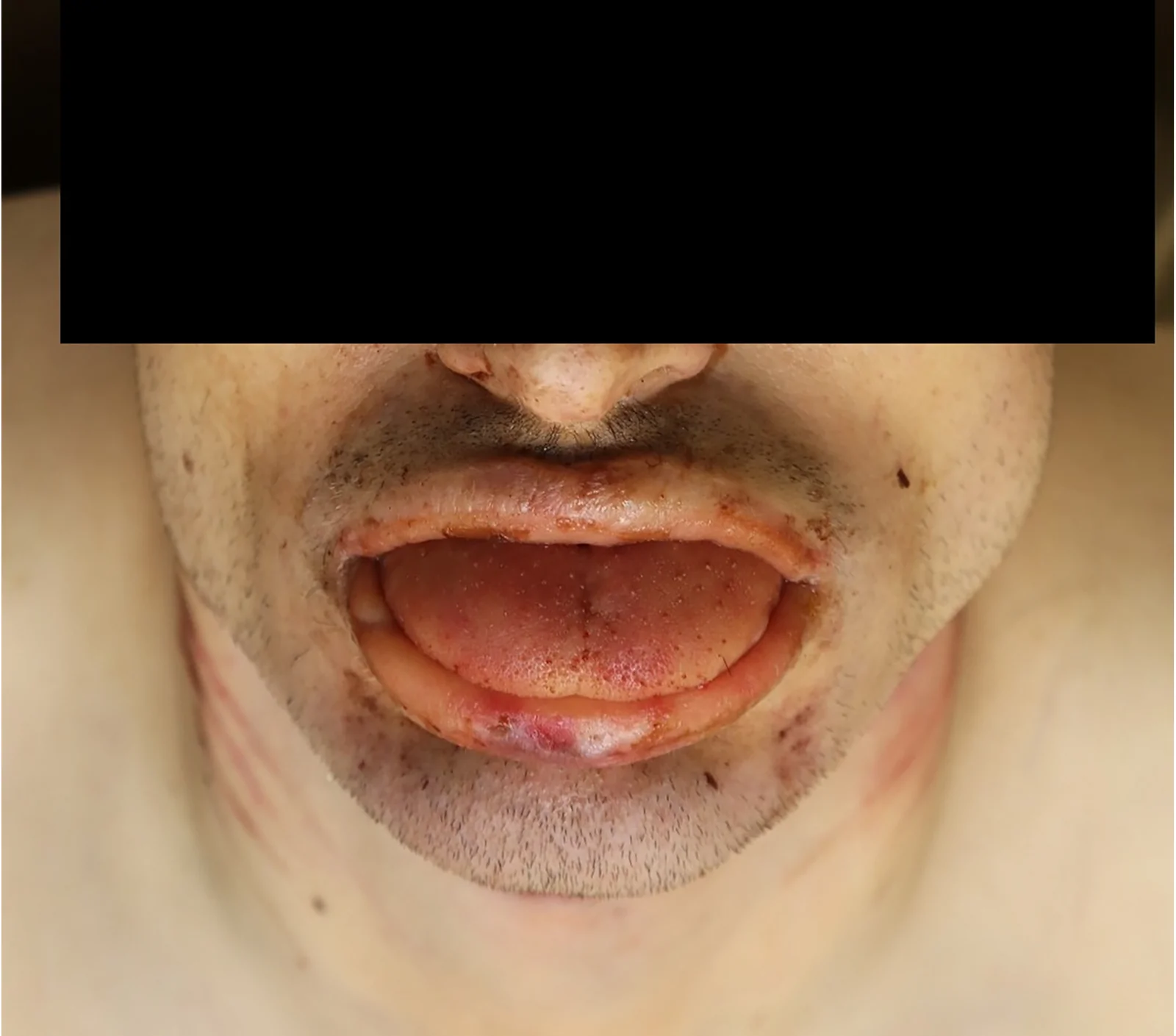
Protuberance of the tongue.

**Figure 5.**
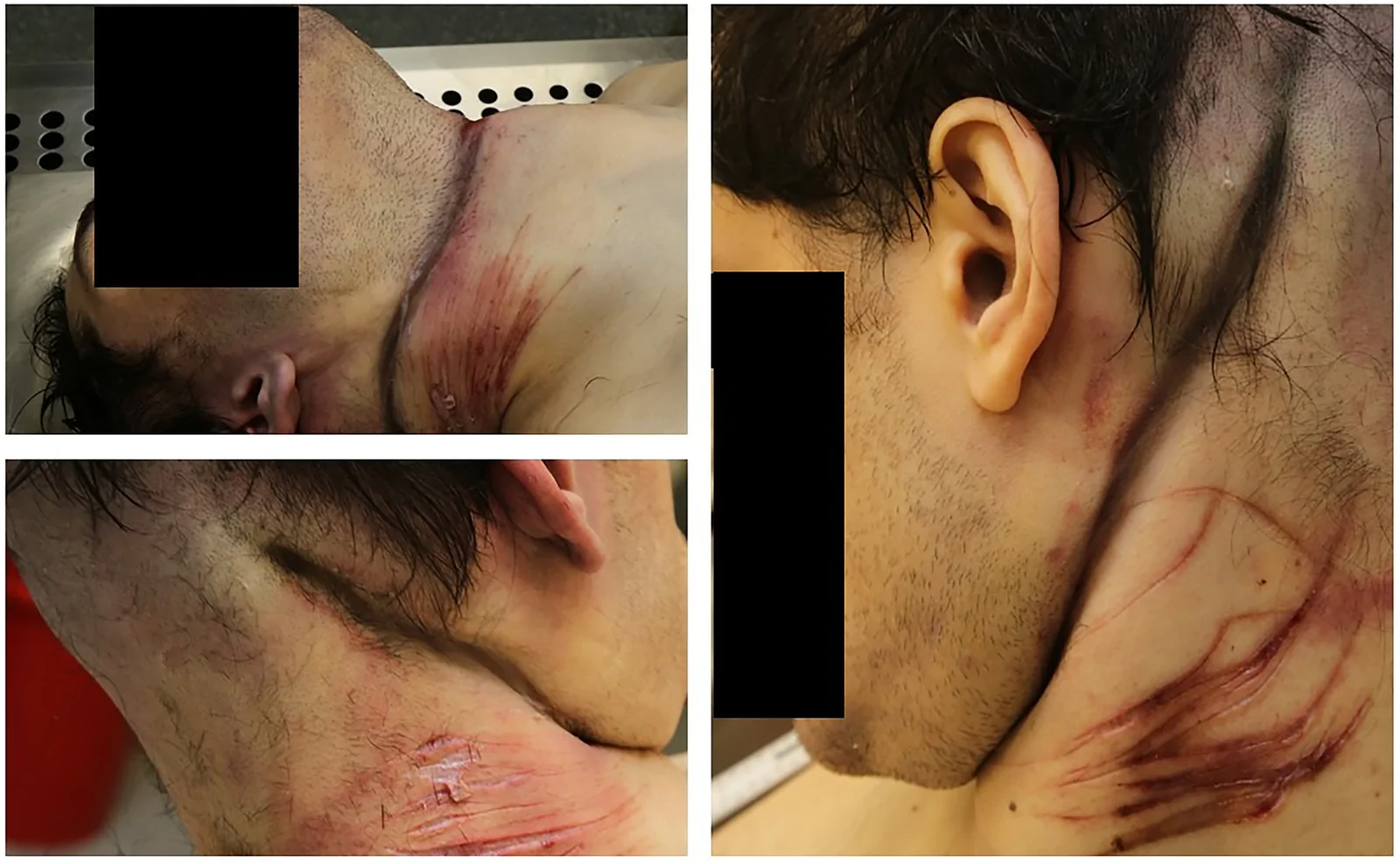
Upwardly slanted and posteriorly incomplete ligature mark. Multiple, superficial, parallel, linear incised wounds of the neck.

**Figure 6.**
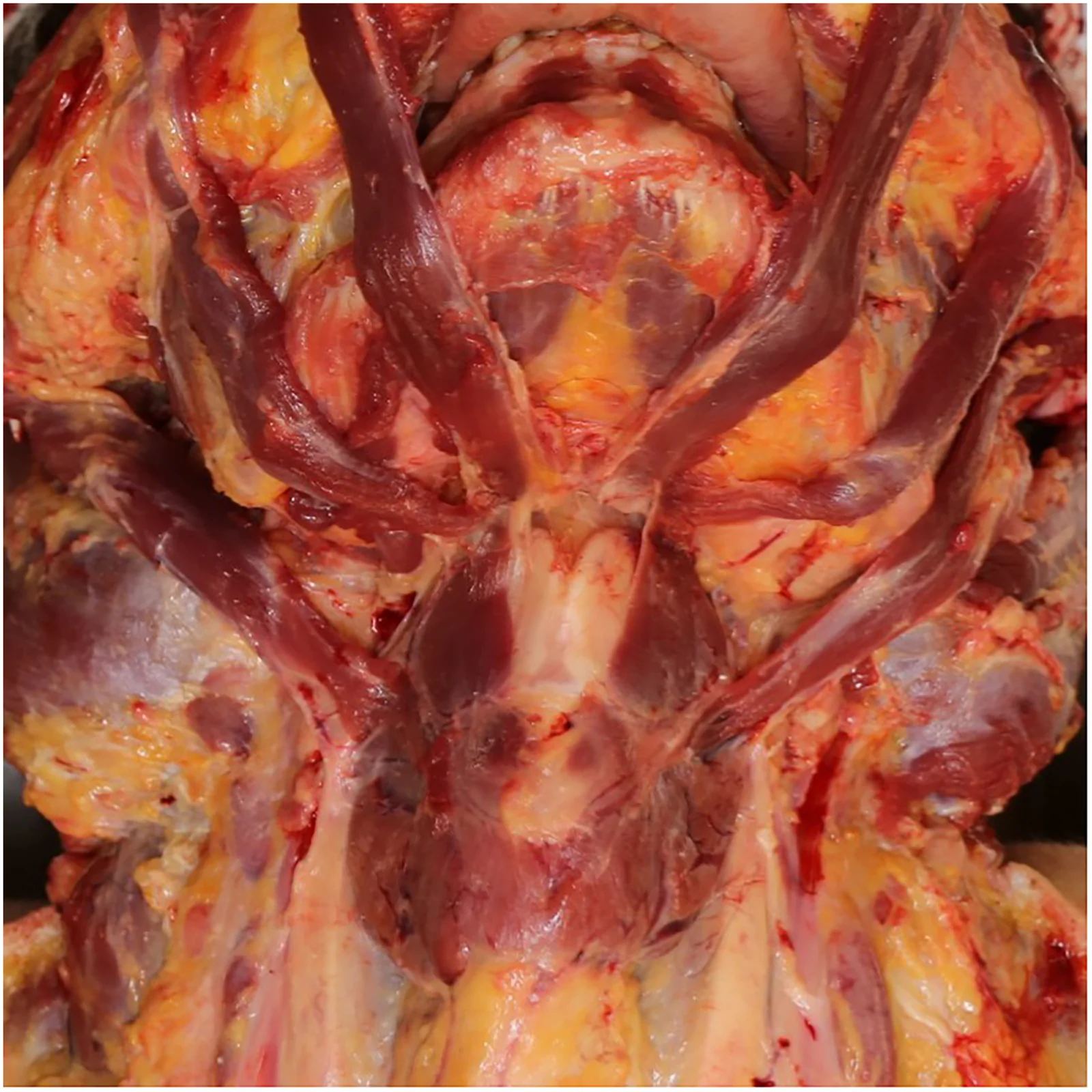
Layered dissection of the anterior neck without contusion of the anterior cervical muscles.

Postmortem examination yielded the body of a slightly overweight young adult man with gravitationally dependent hypostasis within the lower limbs, consistent with the body position when found. There was no congestion or cyanosis of the face, and the eyes were devoid of petechiae. The tip of the tongue was externally protuberant. A torn black plastic garbage bag was over the head. The plastic bag had been torn open on scene by the police to facilitate visual confirmation of identity. The neck exhibited an upwardly slanted, posteriorly incomplete, ligature mark of 1.2 cm width that was consistent with having been caused by the cylindrical electrical cord which had been fashioned into the cervical noose ligature which had been around the neck. Layered dissection of the anterior neck in a bloodless field revealed a fracture of the left superior horn of the thyroid cartilage and bilateral fractures of the greater cornua of the hyoid bone, without contusion of the anterior cervical muscles (Figures 4 to 6).

There were multiple, superficial, parallel, linear incised wounds of the left and right sides of the neck and across the front of the left and right forearms. The incised wounds of the neck had not injured the common carotid arteries, jugular veins, or vagus nerves.

**Figure 7.**
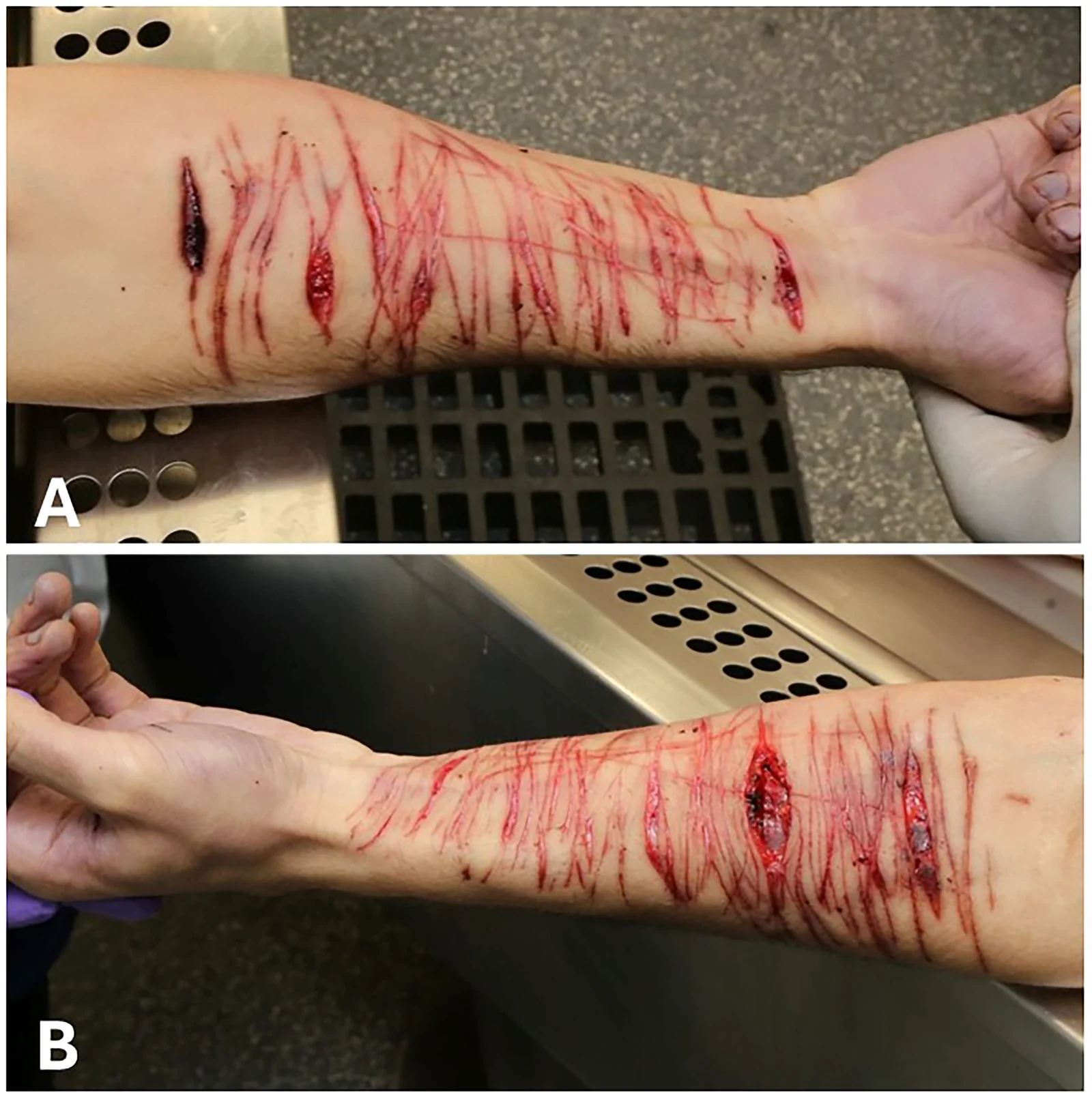
(A) A wide, gaping incised wound across the front of the right wrist. (B) Three deep, linear incised wounds on the left forearm.

Interspersed among the multiple superficial incised wounds across the forearms were three deeper, linear incised wounds of the left forearm of 6.5, 5, and 3.8 cm and a 5 cm × 1 cm gaping incised wound across the front of the right wrist. Solitary, superficial incised wounds were present on the abdomen, front of the right thigh, left hip, and palmar aspects of both hands. Several discrete small abrasions were also noted (Figure 7).

Examination of the major organs was unremarkable. There was no gross or microscopic natural disease that contributed to death. Standard biochemical analysis of the vitreous humor was unremarkable.

Toxicological analysis of the femoral venous blood and urine by a forensic toxicology laboratory reported unquantified detection of psilocin and acetaminophen in the urine as the only positive findings. That laboratory did not possess an analytical method to quantify psilocybin. Attempted analysis of the femoral venous blood by a second forensic toxicology laboratory for psilocybin was unable to produce a result due to analytical issues with the referred sample.

## Discussion

To our knowledge, we report the first case of complex fatal self-harm associated with psilocybin ingestion in an individual with no known prior suicidality. The first method of self-harm that was employed was the infliction of incised wounds to the neck, forearms, and elsewhere. This was followed by the concomitant application of a black plastic garbage bag over the head with occlusion of its mouth around the decedent's neck by an electrical cord as a ligature to effect plastic bag asphyxia-facilitated hanging, with the electrical cord on the external aspect of the bag.

The features of the incised wounds of the neck and forearms indicated self-infliction at sites of predilection for intentional incisional self-harming injury. In terms of chronology, the presence of blood on the in situ electrical cord ligature around the decedent's neck is consistent with the decedent having placed it after he had sustained the incised wounds. Analysis of the urine identified the presence of psilocin; however, it was not possible to quantify psilocybin in the blood. The presence of psilocin in the urine can only be accounted for by metabolism of psilocybin in the blood. The disarray of the scene is in keeping with the psychological effects of psilocybin intoxication.

Complex suicidal (intentional fatal self-harming) deaths can be classified as (i) planned or unplanned or (ii) primary or secondary. Complex suicides are deemed as primary when the self-harming modalities are applied simultaneously or secondary when the self-harming modalities are applied in chronological succession.^13^ The current case describes the use of three modalities that consist of infliction of incised wounds, plastic bag asphyxia, and hanging. The presence of incised wounds prior to completion of the suicide attempt is in keeping with published reports of secondary unplanned complex suicides.^13^ In these cases, it is theorized that excessive pain or the failure of the original method results in a change in suicide modality. Similarly to this case, incised wounds were more frequently followed by hanging.^13^ However, it is worth noting that the combined use of plastic bag asphyxia and hanging possibly represents a planned primary component.^13^ Nonetheless, complex suicides can present a challenge in forensic pathology as they may raise the suspicion of homicide. As reported here, the nature of the injuries at autopsy and the findings of the scene examination confidently establish their self-inflicted nature and chronology of the applied self-harm modalities to effect them.

The decedent was not known for suicidal ideation or prior attempts, in keeping with prior psilocybin-associated case reports.^9,12^ However, psilocybin is generally associated with a reduced risk of suicidal behaviors.^6,14^ A previous survey has shown that the risk of harm increases with higher doses of psilocybin, prolonged duration of the experience, and lack of a supporting environment.^7^ In this case report, the decedent was at increased risk due to the lack of social support during ingestion. Due to the lack of a quantified blood psilocybin concentration, we are unable to ascertain the possible contribution of the dose. The importance of a safe environment with psychological support is often emphasized in clinical studies, but despite this, a clinical trial with treatment-resistant depression reported increased suicidal behaviors and ideation in their high-dose psilocybin treatment arm.^15,16^ Currently, the Canadian Network for Mood and Anxiety Treatments recommends that psilocybin can only be considered an experimental treatment due to a lack of high-quality evidence of its safety and efficacy.^17^

### Manner of Death Considerations

Suicide as a manner of death requires demonstrated intent to fatally self-harm and a standard of proof that exceeds other competing possibilities.^18^ However, fatal self-harming that occurs in the context of drug intoxication without any demonstrated prior intent to take one's life raises the question as to the true underlying reason for the fatal self-harming. Indeed, a previous case report of an individual without a psychiatric history who presented with self-harming behavior in the context of psilocybin ingestion was unable to prove intent of “suicide attempt” upon recovery.^12^

In forensic pathology practice, there are divergent views regarding the role of intent when certifying suicide as the manner of death. Within the framework proposed by the National Association of Medical Examiners (NAME) for manner of death classification, engaging in an inherently dangerous volitional act implies accepting the risk of death as the “sub-intent” of the act.^18^ This perspective allows classification of the manner of death as suicide even when the decedent's state of mind and intent to die are difficult to establish. This scenario is similar to Russian roulette, where the act of placing a loaded firearm to the head is inclusive of accepting a definite risk of death when the trigger is depressed, and deaths occurring in this setting are commonly classified as suicide by convention.^18^

However, when concomitant drug intoxication is present, the general recommendation proposed by the NAME is to classify the death as suicide because the effects of intoxication on judgment and behavior are argued to be inherently subjective.^18^ Analogous scenarios include when suicide is achieved under the influence of ethanol in hanging, descent through a height, or jumping in front of a train.

It has been argued that diminishing the role of intent does not remove the need to infer the decedent's mental state but instead redirects the inference toward volition.^19^ In other words, there remains the need to infer the determination to perform the act rather than the specific reason for the act itself. Therefore, it has been proposed that intent should remain integral to the manner determination of suicide to remain consistent with the term's common meaning.^19^ Although the determination of intent is inherently subjective, it can be inferred through the integration of medical history, circumstances of death, scene investigation, and autopsy findings.^20^ This process is analogous to the clinical judgment used by clinicians when a patient's history is incorporated to rank the most probable differential diagnoses in order of investigative importance. Ignoring such contextual information may therefore be inappropriate, similar to disregarding travel history in a patient presenting with fever that may be infectious in origin.^20^ In the present case, the combination of an absence of established prior suicidal ideation, toxicological evidence of psilocybin ingestion, and the disorganization observed at the scene all support the possibility that the complex fatal self-harming was precipitated by the pharmacological effects of the drug. Despite the volitional act of self-harm, it is difficult to determine the intent of the act, and the terminology “self-harm” has therefore been used preferentially in our discussion instead of suicide.

In Canada, the standard of proof for certification of suicide as the manner of death is grounded in case law^21^ at what is known as the Canadian civil standard of proof and is on the balance of probabilities (more likely than not; greater than 51%) as stated in the NAME Guidelines.^18^ Although this is the required standard, in practice coroners and medical examiners rarely classify a death as suicide unless there is clear evidence of intent. In some jurisdictions, case or other law requires a preponderance of evidence (greater than 70%) to exist for a classification of suicide as a manner of death.^18^

This case was performed in a (medical) coroner's jurisdiction in Canada with the coroner having statutory responsibility for certifying both cause and manner of death. Despite the offered forensic pathology opinion of suicide as the manner of death (akin to an ethanol-intoxicated individual ending their life by hanging or a methamphetamine-intoxicated individual jumping off a building in the belief that he/she could fly), the manner of death was ultimately certified as Undetermined by the investigating coroner following consultation with the regional supervising coroner.

## Conclusion

In summary, we report a case of complex fatal self-harming in the setting of isolated psilocybin ingestion, in an individual who is a regular user of magic mushrooms without a history of prior suicidal ideation or attempts. This underscores the potential harm of psilocybin and the uncertainty of manner of death certification in cases lacking prior demonstrated intent to self-harm.
